# New Methods of Treatment in Erysipelas

**Published:** 1890-11

**Authors:** 


### NEW METHODS OF TREATMENT IN ERYSIPELAS.

1. METHOD OF ROSENBACH.— Consists in first washing with soap not only the affected part, but also surrounding healthy skin, then applying each day a solution of carbolic acid (five per cent.) dissolved in absolute alcohol. Results very brilliant, as regards both the progress of the malady and the febrile phenomena. The use of absolute alcohol by itself has also produced favorable results.

2. METHOD OF NOLTI.— The affected parts and surrounding skin are covered twice daily with mucilage of gum Arabic, mixed with from three to five per cent. of carbolic acid. Good results. Dr. Ebstin mixes the carbolic acid with vaselin.

3. METHOD OF KOCH.— By means of a soft brush we apply a thin and regular covering of the following pomade :

℞—Creolin........................... gramme 1.00.
Iodoform........................... grammes 4.00.
Lanolin............................. grammes 10.00.

The parts are then covered with leaves of gutta-percha. This has given good results, especially in erysipelas of the face and head.

4. Method of Nussbaum and Brunn.— Ichthyol, with or without collodion. Results favorable and very prompt.

5. Method of Hallopeau.— A solution of one to twenty of salicylate of soda is soaked in a mask of several thicknesses of linen and applied over the parts, after which it is covered with rubber bands, to prevent evaporation. Relief almost immediate; cure in from three to five days.

6. Method of Huetter.— Injections of carbolic acid in the healthy skin, in doses of from ten to fifteen grammes, distributed in several punctures, at one or two centimeters from the edge of the affected parts, with the following solution, recently prepared:

℞— Carbolic acid (pure)
Absolute alcohol.................. āā grammes 3.00.
Distilled water.................... grammes 94.00.

Very painful. Only applicable in severe cases of the head and face.

7. Method of Kraske.— Scarify the edges before application of the antiseptic substance. Dr. Lowenstein advises that the incision should be made exclusively in the healthy skin, after which the parts are enveloped with a solution of carbolic acid or sublimate.

8. Method of Wolfler.—Mechanical compression by means of adhesive plaster applied on the healthy skin on the borders of the affected parts, so as to completely surround them.— *Le Bulletin Médicale.*

---

Iodine in the Treatment of Warts.—Given to adults, in doses of ten drops twice daily, the tincture of iodine has proved very successful in preventing the further development of warts and causing those already present to disappear. Dr. Imossi remarked, in the ten cases which had come under his observation, that the treatment produced striking emaciation, a fact which he has since turned to account in the treatment of obesity. He has succeeded in procuring a diminution of weight of about twelve pounds in three weeks.— *The London Medical Recorder.*

